# Enhanced Prediction of Soil Carbon via Encoder-Decoder Neural Networks for a Boreal Study Area in Northern Ontario

**DOI:** 10.3390/s25082583

**Published:** 2025-04-19

**Authors:** Rory Pittman, Baoxin Hu

**Affiliations:** Department of Earth and Space Science and Engineering, York University, 4700 Keele Street, Toronto, ON M3J 1P3, Canada; rpittman@yorku.ca

**Keywords:** soil carbon, digital soil mapping, encoder-decoder, convolutional neural network, quantile mapping, uncertainty maps, boreal forest

## Abstract

Addressing the impacts of carbon in connection with land cover conversion and climate change is of predominant interest for boreal realms. Consequently, boosting accuracy for the prediction of total carbon (C) with soil mapping is a crucial objective, particularly for a boreal study area under risk of land cover transition in northern Ontario, Canada. To enhance the prediction of soil modeling, integrated approaches combining encoder-decoder (ED) with dense neural network (DNN) and convolutional neural network (CNN) formulations suitable for smaller target data sets were developed. These methods were able to effectively extract dominant features within predictor data and augment modeling accuracy. The obtained results were compared with those attained from structural equation modeling (SEM) and random forest (RF), as well as basic DNN and CNN models. A model ensemble based on all approaches was also considered, from which standard deviations were calculated to gauge the prediction uncertainty. Quantile mappings with respect to deciles were also derived from the model ensemble to provide additional insights with prediction. Validation accuracies for the ED-CNN model attained a coefficient of determination (R^2^) of 0.59. The greatest deviations with predicting C contents corresponded to the wetlands. However, when quantified by decile mapping, forested localities within river valleys encountered the highest uncertainties with prediction, indicting a need for better modeling of sites with intermediate concentrations of soil C.

## 1. Introduction

Presently it is well-known that vast stocks of carbon subsist within boreal regions [1,2]. It is anticipated that climate change, in addition with economic prospects, will trigger land cover conversion for the southern transition zone of the boreal forest [3]. This transition is of concern, as this land cover conversion can lead to the loss of carbon sinks within soil and vegetation canopy. Consequently, there is a need to sufficiently quantify the reserves of carbon within boreal regions [4]. It is surmised that within boreal realms, specifically wetlands, that greater reserves of carbon are currently stored within the ground than with the overlying vegetation [4]. To estimate these carbon stocks within the ground, various approaches of soil modeling [4,5,6] can be investigated. Digital soil mapping has evolved into a dominant paradigm for modeling soil properties [7,8], which postulates that soil is modified by soil formation factors [7]. Typically, environment covariates as obtained from remote sensing technologies [8] can be wielded as predictors conforming to soil formation factors, from which models can be fitted for soil carbon. This generally conforms to an end goal of attaining the most possible accurate prediction maps [6] of soil carbon for the targeted study areas. These prediction maps and products can then assist stakeholders with decision analyses to mitigate the potential release of additional carbon from further land cover conversion. Subsequently, there is a need for the accurate estimation of soil carbon and to pursue modeling approaches for which to maximize prediction accuracy.

Machine learning approaches have acquired mainstream adoption within digital soil mapping. Random forest (RF) approaches have been very common [4] due in part to their ability to be applied to both regression and classification [9] and regularly attaining the best accuracies [5,10,11]. Other utilized machine learning approaches include support vector machine [5,12] or additional approaches relating to decision trees [10,13] or k-nearest neighbors (k-NN) [5,10,14]. However, depending on the context, the best modeling accuracies attained from these approaches can be regarded as relatively poor. This is oftentimes the case, even if these accuracies are an improvement from those achieved with conventional regression or classification approaches. Specifically, for studies concerning the boreal forest of northern Ontario, Canada, modeling accuracies for regression of soil carbon have reported coefficients of determination (R^2^) less than 0.30 [14,15]. The models for these noted accuracies corresponded to RFs and k-NNs. However, even for other targeted soil properties in different regions around the world, realized modeling accuracies can be augmented as R^2^ values less than 0.3 have been reported [4,6,16]. Therefore, improving modeling accuracy is a current topic of investigation for digital soil mapping and thus an active research pursuit.

Deeper learning methods, generally involving neural networks (NNs), have lately been gaining popularity within digital soil mapping. These can comprise simpler NNs composed of dense layers, here known as dense neural networks (DNNs); these have been identified also as multilayer perceptron (MLP) NNs [10] or artificial neural networks (ANNs) [5,12] in the literature for soil mapping research. DNNs are constituted of one or more hidden layers of interconnected units with weightings iteratively adjusted via an intrinsic learning process [5] and have been trained for classification [5,10], as well as regression [12,17]. However, DNNs investigated within digital soil mapping have generally only attained modeling accuracies comparable to RF approaches [5,10]. As a next step for incrementing NN complexity, research in other domains has pursued convolutional neural networks (CNNs) [18], particularly regarding image classification [19,20]. CNNs contain feature extraction and selection layers, in addition to dense layers like those within DNNs [18,21]. Many refined formulations and architecture of CNNs have been proposed and predominantly achieve superior modeling accuracies [18]. However, these models require the optimization of tens of thousands, if not millions, of parameters [18]. Correspondingly, to prevent overfitting or overgeneralization, these models require sufficient quantities of target data. Within soil sciences, it is simply not feasible to attain adequate amounts of soil samples necessitated for training larger CNNs. Many soil modeling studies are typically confined to fewer than a few hundred sampling sites [4], which limits data that can be exploited for model training and subsequent validation. This arises mainly due to the expense and difficulty of conducting field campaigns, especially in remote localities, for obtaining the necessary quantities of samples for ground truth. Therefore, the curtailed sample sizes with soil modeling manifest as a bottleneck for the adoption of CNNs.

Encoder-decoder NNs are an adaption of NN structure integrated within encoder-decoder (ED) methodology [22,23]. Recently, EDs have demonstrated success in augmenting accuracies with regression modeling for soil moisture [24,25] and temperature [26] applications, as well as for digital soil mapping [27] for a larger open dataset. The architecture of EDs is first composed of an encoder, which condenses information from an original input to a compressed representation [22]. From there, a decoder can reconstruct details [22], from which regression or classification can be implemented. In essence, an ED can filter out noise from the original inputs [22], leading to the retention of information more relevant for modeling purposes. In this context, the ED is considered as a feature extraction, with raw data as input. This is expedient for digital soil mapping as large sets of environmental covariates are usually wielded as predictors, even if fundamentally only smaller subsets are relevant for prediction [6]. Within the encoder and decoder formulations, various types of architecture can be implemented. These can conform to simple or sparse layers, deeper DNNs [28,29], or CNNs [23,27] including fully convolutional networks (FCNs) [22]. Furthermore, EDs can also be configured to consist of NNs with reduced layers and units [30], permitting adaptability to smaller sets of data such as encountered in digital soil mapping research.

Prediction maps are often a deliverable of digital soil mapping, with the objective of attaining the most accurate predictions possible. In addition to improving accuracy, there is also the motivation of quantifying some form of uncertainty [4] with modeling prediction. An ensemble of predictions [31,32] from the best models corresponding to a variety of modeling approaches can be harnessed for resolving uncertainty of prediction in terms of standard deviations. In addition, a mean value of prediction can be calculated from this ensemble. It is likely that the best prediction would still be obtained from just the one most accurate model, but nonetheless, this average prediction with the ensemble could be regarded as more robust [31]. Investigating various modeling approaches with an ensemble can corroborate whether separate models underpredict or overpredict comparably regarding residuals. As well, resolving uncertainty with prediction can lead to insights into what localities within a targeted study area have the greatest uncertainties with prediction. To address overprediction or underprediction of a soil property, quantile mapping [33] via deciles of prediction quantities can be examined. To the best of our knowledge, this has not been considered within digital soil mapping. Moreover, the resulting standard deviation of the deciles from an ensemble can wield further information into deciphering what land cover types conform to the greatest prediction uncertainties.

In summary, the main aims of this study were to develop ED approaches to improve the estimation accuracy of soil properties with smaller sets of target data and to gauge the uncertainty of prediction based upon an ensemble for standard deviation decile maps. EDs were trained to model soil carbon for a study area of the boreal forest in northern Ontario, Canada. These results were compared with those attained from other modeling approaches, consisting of RF, structural equation modeling, DNN, and CNN. A model ensemble based upon all approaches was then compiled, from which an average and uncertainty of predictions as by standard deviations were computed. This was followed by quantile mapping with respect to deciles, which also evaluated for uncertainties with predictions that provided effective insights into the modeling with respect to land cover.

## 2. Materials and Methods

### 2.1. Study Region

**Figure 1:**
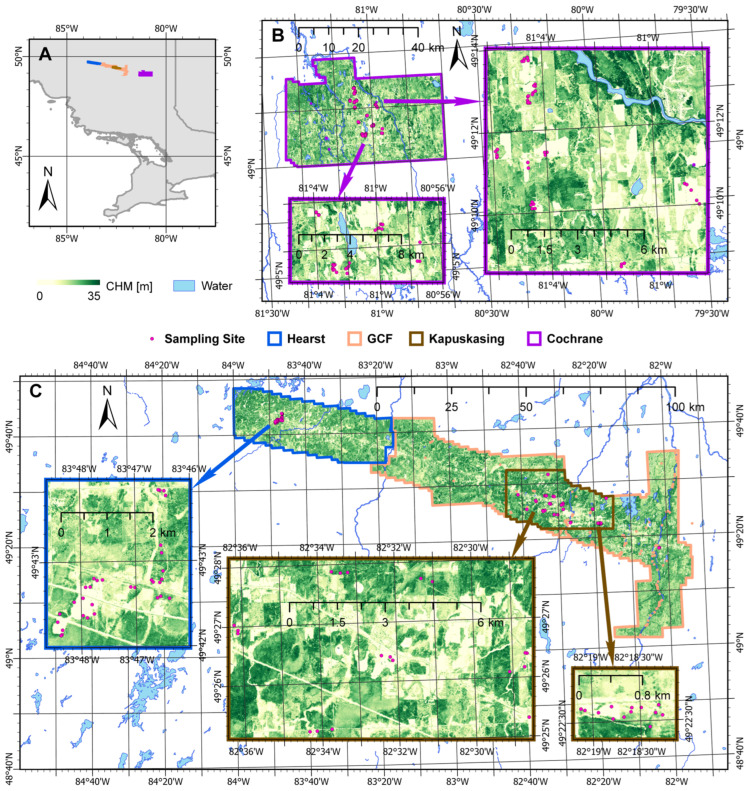
The study areas in northern Ontario, Canada. The backgrounds for the designated study areas within the subfigures that indicate the locations of sampling sites correspond to a canopy height model (CHM) [m]. Also depicted are rivers and lakes within the study region. (**A**) Location reference of the study region within Ontario, Canada. (**B**) Location of sampling sites within the Cochrane study area, with insets focused on clusters of sites. (**C**) Location of sampling sites within the Hearst, Gordon Cosens Forest (GCF), and Kapuskasing (within the GCF) study areas, with insets focused on clusters of sites.

The study region corresponded to the southern transition zone of the boreal forest within the District of Cochrane, in northern Ontario, Canada. The designated study areas centered upon the Ontario highway 11 corridor and were located from Hearst in the northwest to the community of Cochrane in the southeast (80°40′ W–84° W, 49° N–50° N). These study areas consisted of Hearst, the Gordon Cosens Forest (GCF), Kapuskasing within the GCF, and Cochrane. The locations of the delineated study areas, as well as locations for soil sampling sites, are shown in Figure 1. Soil sampling sites are indicated, which amounted to a total of 144 sites. These consisted of 32 sites within the Hearst area, 42 sites near Kapuskasing with 3 sites near Fauquier-Strickland within the GCF area in the center of the study region, and 67 sites within the Cochrane study area. The availability of light detection and ranging (LiDAR) retrievals delimited the areas encompassed by the study areas as the bounds of the study areas coincided in part to where LiDAR data [34] were obtained. Within Figure 1, the background for each study area corresponds to a canopy height model (CHM) of 10 m spatial resolution. This CHM was derived from the LiDAR data, which were collected during the autumns of 2016 and 2017 for the Ontario Ministry of Natural Resources (MNR).

This region was part of the Canadian Shield, conforming to topography that was relatively flat at the landscape scale. Elevations ranged from a minimum of around 120 m in the northeast to about 350 m northwest of the community of Cochrane, with some local variation of topography in this locality. Overall, elevations gradually increased by around 100 m toward the westernmost study areas. The climate throughout the study region was homogeneous [35,36,37], and land cover was mostly forested with agriculture activity present around the communities along the Ontario highway 11 corridor. Common endemic tree species for this region were black spruce (*Picea mariana*), balsam fir (*Abies balsamea*), trembling aspen (*Populus tremuloides*), and balsam poplar (*Populus balsamifera*). Other subsisting tree species included white spruce (*Picea glauca*), tamarack (*Larix laricina*), and less frequently eastern white cedar (*Thuja occidentalis*). Due to the presence of luvisol deposits composed of high clay component at the deeper soil horizons, jack pine (*Pinus banksiana*) was essentially absent within these defined study areas. The idiosyncratic deposits of gray luvisols underneath the surface layers throughout this expanse have earned this region the denotation as the Great Clay Belt of northern Ontario. In addition to luvisols, other soil types present in this study region included gleysols and mesisols within the environs of the wetlands [38].

**Figure 2:**
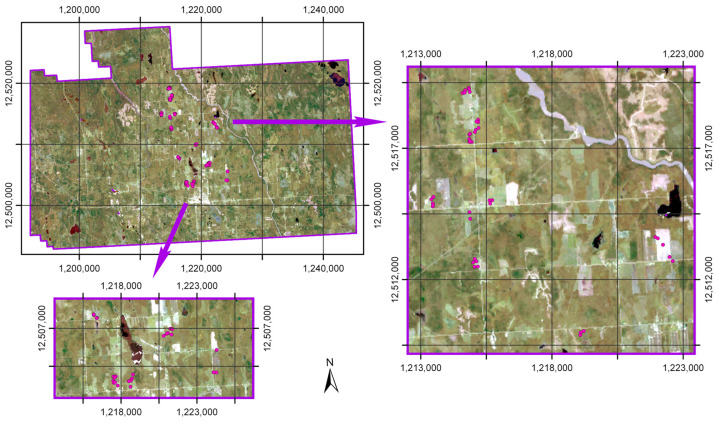
True-color composite Landsat-8 image compiled for summer (June–August) of 2017 for the Cochrane study area, with sampling sites indicated (i.e., pink dots). Insets focused on clusters of sampling sites (following from the arrows). Coordinate grids are in the NAD 1983 Lambert conformal conic projection, with eastings [m] and northings [m] marked.

Of interest for this research was prediction within the Cochrane study area (80°40′ W–81°25′ W, 49° N–49°20′ N). Specifically, the Cochrane study area was selected due to its location within the southern transition zone of the boreal forest and greater economic pressures pertaining to agriculture that can lead to land cover conversion. A true-color composite of the Cochrane study area as compiled from Landsat-8 imagery for the summer (June–August) of 2017 is depicted in Figure 2. The Cochrane study area consisted of wetlands along its western delineated edge, as well as within its northeastern portion. Agricultural lands composed the center of the study area. The Abitibi River transversed through the eastern central part of the study area along a northwest trajectory.

### 2.2. Soil Data

Soil samples were collected for a total of 144 sites, which were extracted during field campaigns in September 2018 (12 sites), August 2019 (28 sites), and August–September 2021 (104 sites). Each of these sites were composed of three subplots, located within 2 m radii at distances of 4.5 m, 7.5 m, and 9.5 m from the site center at bearings of 0°, 120°, and 240°, respectively [39]. In total, soil samples were acquired from 431 subplots, with the exception of 1 subplot for the September 2018 field campaign. These samples were obtained from land covers representative of the study region, corresponding to forested, agricultural, and wetland cover types. The dominant tree species present were recorded for each site. When possible, a few sites in close proximity to one another, but each conforming to a different land cover type, were sampled [40]. The sites were located far enough away from one another to correspond to different pixels of rasterized remote sensing imagery, as harnessed for environmental covariates. An inferential statistical analysis confirmed that an adequate number of sites were sampled to capture the heterogeneity across the various land cover types present in the study region [40].

Soil samples for both chemistry and bulk density analysis were obtained from the subplots. The investigated soil property was total carbon (C) with respect to concentrations (i.e., [%]) in samples collected at the 5–15 cm depth layer of mineral soil. This depth was selected in part as this was typically the layer with maximal C concentrations, as well as due to biological relevance with the presence of roots. These concentrations of C were resolved by combustion methods and were retained in the original units as measured within the laboratory. Other studies within the boreal forest of Canada have also regarded soil C in concentrations [2,40]. The concentration value of C per site was calculated as the average of C concentrations among the subplots within each site. These soil samples were analyzed at the Great Lakes Forestry Centre (Sault Ste. Marie, ON, Canada) for the samples collected during 2018 and by A&L Canada (London, ON, Canada) for the samples retrieved during the 2019 and 2021 field campaigns.

### 2.3. Environmental Covariates

A variety of environment covariates conforming to soil formation factors were wielded as predictors for the modeling of soil C. These environment covariates were obtained from a few different remote sensing technologies. In total, 34 environmental covariates were employed, relating to the soil formation factors [8] of topographic relief, vegetation, parent material, and time. The spatial resolutions of the utilized environmental covariates were principally 30 m; aeromagnetic data and the black spruce indicator were resampled to 30 m spatial resolution. A complete listing of the environmental covariates, along with the sensor source and relating soil formation factor, is presented in Table 1. The seven environment covariates denoted with an asterisk (*) were the higher-ranked predictors resolved for structural equation modeling (SEM) (discussed later).

A digital elevation map (DEM) was derived from LiDAR data [41] from which a suite of topographic covariates was computed via SAGA (System for Automated Geoscientific Analyses) GIS (geographic information system) version 7.6.3 software [42]. In total, 15 environmental covariates were generated from the DEM; these are listed accordingly in Table 1. The LiDAR data were retrieved by aerial survey during the autumns of 2016 and 2017, with a point density of approximately eight retrievals per m^2^ [34]. Also generated from the LiDAR data was a canopy height model (CHM), calculated as the height of the canopy layer above the DEM; this was computed as the difference between a digital surface model and the DEM. A gap fraction, here defined as the portion of retrievals with only one return, was also calculated [41]. Regarding interpretation, the gap fraction was highly correlated with CHM. However, the gap fraction encapsulated vegetation density for canopies that were not necessarily taller, yet had denser structure as encountered in some forested environments.

Surface reflectance (SR) was obtained from Landsat-8 imagery for summer (June–August), the season of peak vegetation, for the study region for 2017, that is, for the year that the latest LiDAR data were retrieved. This SR was compiled from median pixel intensities over the duration of the defined summer season, from imagery scenes with less than 2% cloud cover. From that SR, the normalized difference vegetation index (NDVI) and modified normalized difference water index (MNDWI) were each calculated. Landsat-5 imagery was also acquired, from which a change detection magnitude was generated. This was calculated as the Euclidean norm of the differences between SR for red, infrared, and shortwave infrared bands, between 1984 and 2005. For this study region, during that time interval, there were considerable land cover conversion changes regarding agricultural sites as some cropland was left fallow and subsequently abandoned or converted to pasture. This ensuing land cover transition with vegetation related to C sequestration [43].

Environmental covariates corresponding to C-band synthetic aperture radar (SAR) were also obtained. These were acquired for the vertical–horizontal (VH) and vertical–vertical (VV) polarizations for May of 2017. The month of May was selected in part because for this study region, the local water levels were then typically maximal due to the completion with melting of the snowpack accumulated during the wintertime. Aeromagnetic retrieval data were obtained from Natural Resources Canada (NRCan), here consisting of magnetic residual from November of 2018 and gravity anomaly from 2016. An indicator for black spruce (*Picea mariana*) was also considered; this was obtained from the National Forestry Inventory (NFI) for the reference year 2011 and generated from a k-NN model fitted on MODIS (moderate resolution imaging spectroradiometer) imagery [44].

### 2.4. Modeling

ED structures incorporating NN components were the focus of the modeling work. However, a variety of modeling methods were explored and implemented for the regression of soil C. These included basic DNN and CNN models, as well as RF and SEM. Formulations for the ED approaches are outlined in a following subsection and briefly discussed in the supplementary models, from which prediction maps were also obtained. Model evaluation for comparing results between the approaches has also been discussed.

Stratified random sampling with respect to the targeted soil property of C was implemented. This was enacted to separate the data into balanced training and validation sets regarding C measurements. Specifically, this was to ensure that both the training and validation sets had commensurate representations of sites with varying C concentrations conforming to different land cover types [40]. For the stratified random sampling, the data were split 70:30 between training and validation, respectively, regarding the sampling site. Accordingly, this stratified random sampling assigned 103 sites for training and 41 sites for validation.

#### 2.4.1. Normalization

Before the fitting of the ED and NN models, the predictor data needed to be standardized via normalization and then split into training and validation sets. The normalization was accomplished by converting all predictor values into associated z-scores [18], using the calculated means and standard deviations per each respective predictor across all sampling sites. For models comprising DNNs, the predictor input was arranged in matrices with the columns pertaining to predictors and rows corresponding to sites. Regarding models consisting of CNNs, the predictor inputs were reshaped into arrays as for the DNNs but with an additional dimension for the one-dimensional (1-D) convolution. Further details concerning other investigated normalization methodology will be expressed in the Discussion Section.

#### 2.4.2. Encoder-Decoder Neural Networks

**Figure 3:**
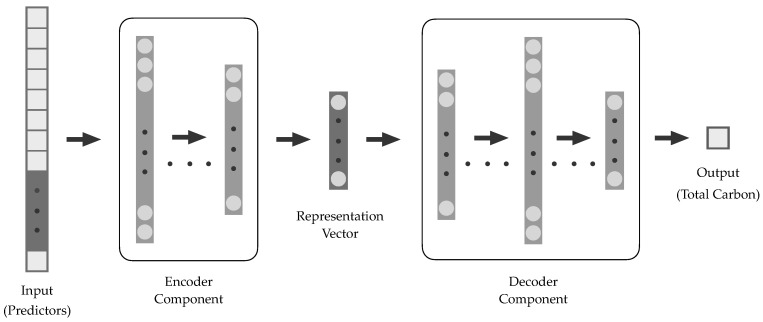
Modeling implementation with encoder-decoder (ED) neural networks.

The EDs each consisted of separate encoder and decoder components, with the ED process illustrated in Figure 3. Formulations implemented for the ED-DNN and respective ED-CNN are outlined in Table 2. For these, the encoder processes each started with an input layer containing predictor information, which was converted via the encoders to representation vectors. These representation vectors were then inputted into decoders, from which output sequences corresponding to regressed soil C concentrations were generated. An ED-DNN was first investigated, followed by a corresponding ED-CNN.

Accordingly, the ED-DNN was composed of dense layers, analogous to a DNN. Within the ED here, the structure for its DNN encoder was basic, with just two dense layers. The encoder finished by condensing the information to a representation vector of eight units, which was the subsequent input for the decoder part. Likewise, the DNN decoder just consisted of three dense layers, with an upscaling exerted before the final downscaling. This was implemented as this architecture for the ED-DNN could remove correlation among predictors and extract the key representation of the variables [29]. It was reasoned that eight units for a representation vector were sufficient for condensing relevant information within the predictors [30] for modeling for C.

An ED-CNN was also trained, which consisted of 1-D convolutional layers [30]. In comparison with the ED-DNN, the convolution layers for the ED-CNN supported the resolving of features at additional scales [22]. The CNN encoder consisted of two 1-D convolutional layers, the first followed by a max pooling layer and the second by a flattening layer. As for the ED-DNN, the encoder finished by condensing information to a representation vector of eight units, which was the subsequent input for the decoder. With the CNN decoder component, it was upscaled to a dense layer of 64 units and then reshaped to an array of one row with 64 columns. This was followed by two 1-D convolutional layers, with an upsampling layer of size 2 after the first convolutional layer. After the last convolutional layer, the output was flattened and then succeeded by a dense layer before the final output.

Keras version 2.15.0 [45] in R version 4.4.0 was employed for training the EDs and subsequent DNN and CNN models. These models were fitted for the regression of soil C [%], so all the ending components for the NN structures were formulated with a final output layer of one unit. Rectified linear unit (ReLU) activation functions were specified as these were of a form with sparse activation that tended toward better gradient propagation without a vanishing gradient [18]. The number of units per layer were selected to be multiples of the typical base 2 [18,30,46], with the number of units for initial layers chosen to be less than the number of predictors. As the NNs performed regression, an Adam optimizer [46] with the loss function corresponding to the mean squared error (MSE) was implemented. The NNs were trained to minimize MSE over 5000 epochs; this was deemed a suitable number of epochs for attaining greater accuracies from training the CNNs [47,48] but without overfitting. Epochs of 500, 2000, and 10,000 for model training with the NNs were also investigated. In general, there was not much difference between modeling accuracies when training from 500 to 5000 epochs, particularly for the NNs composed of dense layers. However, accuracies tended to degrade on the validation set for 10,000 or more epochs. The networks composed of CNN architecture required more than 500 epochs of training in order to minimize MSE, hence the selection of 5000 epochs for training with the NN models.

#### 2.4.3. Other Models

For the supplementary modeling, this commenced with the utilization of NNs composed of basic architecture. The objective was to formulate simpler NN structures, to minimize the number of parameters to train. Specifically, this was implemented to mitigate the prospect of overfitting with the modeling [18]. Once the NNs were trained, both an RF and an SEM were independently fitted for comparison purposes. RF models have been popular within digital soil mapping [4] as RF is regarded as a standard approach for both classification and regression modeling [9]. Details of the configurations for all these models are briefly discussed in the following paragraphs.

For the NNs, a DNN of rudimental structure was formulated and was composed of only two internal dense layers. The first dense layer consisted of 32 units, with a subsequent dropout of 0.25; this was followed by a dense layer of 16 units, then a dropout of 0.25, and then the final layer of 1 unit. To improve the modeling accuracy, dropout was implemented twice; this was performed to prevent modeling overfitting [18,49] to reduce the degrading of modeling accuracy. The DNN was regarded as a direct comparison with the RF and SEM as it was comparable with conventional regression approaches with how it handled nonlinearity [12]. A CNN was also investigated. This CNN consisted first of a 1-D convolutional layer of 32 units, followed again by a 1-D convolutional layer of 32 units. This was then succeeded by a 1-D max pooling layer, dropout of 0.25, a subsequent flattening layer, a dense layer of 16 units, dropout of 0.25, and then the final dense layer of 1 unit. For the convolutional layers, the kernel size was set as 1. The input layer for the DNN corresponded to the number of predictors, whereas for the CNN it conformed to an array of one row with the number of columns equal to the number of predictors. For the CNN, the ordering of features can have an impact as for convolution the neighboring pixels were imputed [18]. The predictors were ordered in sections conforming to the sensor source and soil formation factor. In contrast to the DNN, the CNN was better suited for exploiting the nonlinear relationships among the predictors.

An RF and SEM were both trained and evaluated for comparability. The RF was modeled on the same training set of sites and predictors, as performed for the NNs. For configuration, the RF was fitted with 1000 trees. As well, the corresponding number of variable splits (m_try_ parameter) was set to the floor of the square root of the number of predictors, which here was five. The randomForest package version 4.7.1.1 [50] in R was employed for the RF modeling. For brevity’s sake, specific details for the SEM will not be discussed here. The SEM was obtained from a model for total soil C based on a set of what were assessed as the seven most contributing predictors regarding a feature selection (these seven predictors are indicated by asterisks in Table 1). This SEM was trained and then evaluated on the same sets of data by sampling site as performed for the other models. Maximum likelihood estimation was adopted for fitting the SEM, with the parameters resolved by nonlinear minimization subjected to the box-constrained optimization [51]. The lavaan package version 0.6.18 [51] in R was utilized for training the SEM.

Once all the models were trained and evaluated, an ensemble based on all the predictions was also computed. This corresponded to the average (AVG) of the six models: SEM, RF, DNN, CNN, ED-DNN, and ED-CNN. The model ensemble was also considered as specific models yielded advantages for predicting C for certain localities and as a measure to improve robustness [31] and not rely on simulations for one type of modeling. An ensemble composed of the average from all models was also reckoned due to the standard deviations constituting a measure of uncertainty with prediction; this is discussed further in the Uncertainty Quantification Section.

#### 2.4.4. Model Evaluation

All models were assessed on the respective validation set. The stratified random sampling allocated 103 sites for the training set and 41 sites for the validation set. Metrics for evaluating accuracy were the coefficient of determination (R^2^), root mean squared error (RMSE), and mean absolute error (MAE). The R^2^ corresponded to the amount of variation within observations that were quantified by the model [52]. It was calculated as 1 minus the fraction composed of the numerator equal to the residual sum of squares and the denominator as the total sum of squares [52]. MSE and MAE corresponded to the unit of the target variable, C, which were expressed in the same measurements (i.e., [%]). The reporting of R^2^ has been customary for regression [12,52] and was regarded as the primary metric of model assessment; this was done particularly to compare with other modeling approaches such as RF and SEM. Normally, R^2^ is bounded between 0 and 1, with higher scores indicating better accuracy [52]. However, especially for overfitted NNs, negative R^2^ values can be attained upon assessment of the validation set. Negative R^2^ values have been encountered when the residual sum of squares is greater than the total sum of squares; this can occur when models are assessed on validation data that are too dissimilar from the training data used to fit a model [52] or when models are overfitted on the training data. Consequently, to avert overfitting was the main reason why dropout was implemented within the DNN and CNN model formulations.

Error patterns between the various approaches were investigated, in terms of pairwise scatterplots and correlations between the models. If the residuals between two models exhibited high pairwise correlations, this would indicate that the models displayed similar trends with prediction. The model ensemble was formulated so that it could capture some prediction power from each model, with the hopes of constructing a more robust model, even if slightly less accurate than that from the best approach. Scatterplots corresponding to the best modeling approach regarding accuracy were also investigated. This was conducted to acquire a representation of tendencies with the regression, regarding overprediction or underprediction in comparison with measurements.

### 2.5. Uncertainty Quantification

An advantage of obtaining prediction maps from the various models was that a measure of uncertainty was quantified from the discrepancies with prediction. Therefore, gauging uncertainty from a model ensemble was considered. This corresponded to calculating the standard deviation per pixel of a prediction map, based on the predictions from each of the modeling approaches. As the prediction maps each conformed to the same bounded study area, all the prediction maps consisted of the same number of data points. It was ascertained that all prediction maps corresponded to the same projection, registration grids, cell sizes, and spatial extents, with the same counts of non-missing data points. Each prediction map encompassed the same land cover types, meaning each land cover was represented by the same portion of pixels with each prediction map. For that reason, quantile mapping regarding calculated quantiles assisted with resolving if the same localities or land covers were equally predicted, when accounting for model overprediction or underprediction over the range of values.

Quantile maps [33] with respect to calculated deciles were implemented for each model. As a first step, all n data points from a prediction map were sorted by ascending order, i.e., as $x_{1},x_{2},x_{3},\cdots ,x_{n-1},x_{n}$ with $x_{1}\leq x_{2}\leq x_{3}\leq \cdots \leq x_{n-1}\leq x_{n}.$ The number of data points amounted to the number of pixels populated with values, i.e., non-missing. Thus, the corresponding index $i_{q}$ for the q-th k-quantile, 1≤q≤k, q,k both integers, was equated by

(1) $$i_{q}=\frac{q}{k}\cdot \left(n+1\right).$$

To compute the quantile value from its respective index, unless the index was an integer, interpolation was necessary. For the index $\;i_{q}$ equal to a rational number of form x.p, with integer component x and decimal component p, i.e., 0≤p<1, the cutoff of the data value for the qth quantile was calculated as

(2) $$x_{i_{q}}=\left(1-p\right)\cdot x_{\mathrm{floor}\left(i_{q}\right)}+p\cdot x_{\mathrm{floor}\left(i_{q}\right)+1}\;.$$

Here, the floor function returned the integer value of the index, i.e., the greatest integer less than or equal to $i_{q}$. Cutoffs for all k quantiles were calculated accordingly. After that, all data points from the prediction map were binned into their respective quantiles.

In terms of quantiles, it was decided that 10 quantiles, i.e., deciles, were sufficient. An adequate number of quantiles were to be specified, providing resolution to represent the different land cover types present within the study region. Decile maps for each prediction map were computed, by application of the ntile function within the dplyr package version 1.1.4 [53] in R. Pixels with missing data, i.e., data with no values as they corresponded to exterior pixels or gaps within the study area bounds, were excluded from the decile calculations. As for the standard deviation map of predictions for C in original units [%], a map corresponding to the standard deviation of the deciles maps was also generated. This map conformed to a measure of uncertainty when the effects of overfitting and underfitting were mitigated, adding insights to assist with resolving uncertainty with prediction.

## 3. Results

### 3.1. Modeling Accuracies

A DNN, CNN, ED with DNN structure, and ED with CNN structure were all modeled and fitted on the same training data. For comparison purposes, an RF and SEM were each also modeled. Once trained, these models were then employed to predict soil C on the same separate validation set of data. The accuracy results of these models, assessed on the same validation data sets, are reported in Table 3. Also reported is an ensemble of all these approaches, which here constituted the average (AVG) of predictions from all the other models. As observed in Table 3, similar accuracies were attained by the SEM, RF, and DNN. In comparison, the CNN model obtained improved accuracy. However, the EDs attained the best modeling accuracies, with reported R^2^ exceeding 0.50. The ensemble model (AVG) obtained a relatively reasonable R^2^ value of 0.50. In addition, reported RMSE and MAE were also lowest for the EDs.

**Figure 4:**
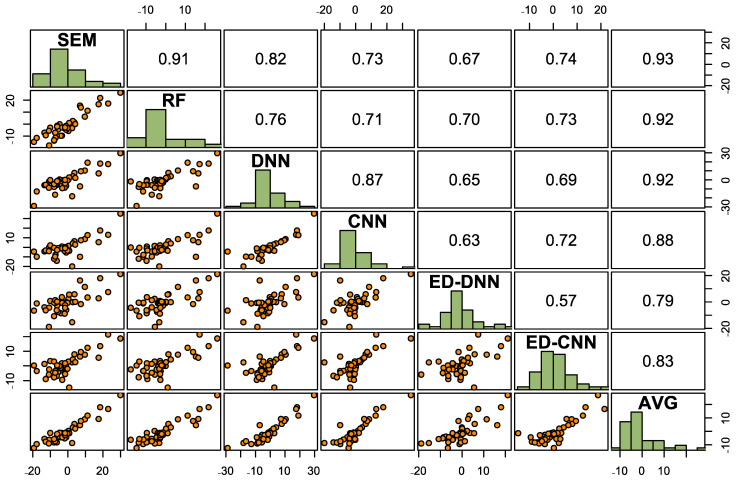
Lower left: Pairwise scatterplots of residuals between models. Diagonal: Histograms of residuals for each model. Upper right: Pairwise correlations between residuals for each model comparison.

The residuals, here defined as the measurements minus respective predictions, were calculated on the validation set for each model. Scatterplots of pairwise comparison between the residuals for each model were generated, with pairwise correlations noted, as shown in Figure 4. Large correlations of greater than 0.7 were reported for most pairwise comparisons, with all correlations greater than 0.5. The residuals for each model were positively correlated with one another. This indicated that the models predicted C with similar tendencies for the various sites, even if the magnitudes of the predicted values differed.

**Figure 5:**
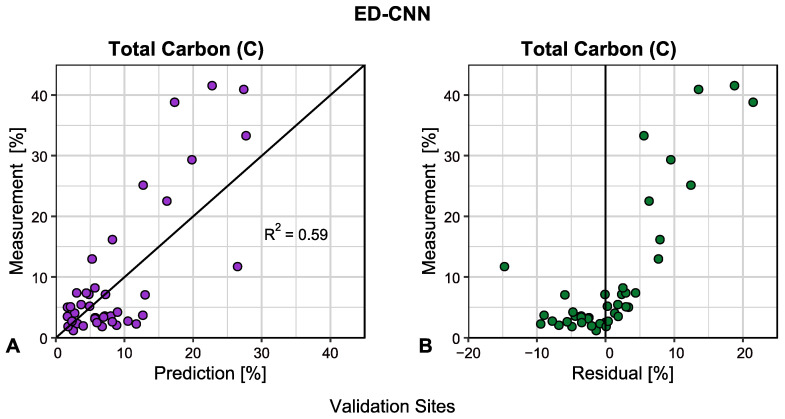
Scatterplots of measurements versus prediction for total carbon (C) [%] on validation sets for (**A**) the encoder-decoder composed of 1-D convolutional neural network layers (ED-CNN) and (**B**) measurements versus residuals for ED-CNN.

A scatterplot of measurements versus prediction for the best model, i.e., the ED-CNN, is displayed in Figure 5. Also depicted in Figure 5 is the scatterplot for measurements versus residuals for the ED-CNN model. For reference purposes, a line corresponding to the measurement equal to the prediction [27] was plotted for the prediction scatterplot, whereas a vertical line denoting a residual value of zero was indicated for the residual scatterplot. As noted, this model obtained a R^2^ value of 0.59. It was observed within these scatterplots that the greatest variations of prediction coincided with the sites of greater soil C concentrations. Soil C tended to be overpredicted for sites with the least amount of C, which for this study area generally corresponded to agricultural fields. However, conversely, these models tended to underpredict for sites with the most C, which for this region conformed to the wetlands. For that reason, quantile mapping regarding deciles were computed to assist in reducing the effects of overforecasting and underforecasting soil C for certain land cover types, motivating the investigation of insights with uncertainty mapping presented later.

### 3.2. Prediction Maps

**Figure 6:**
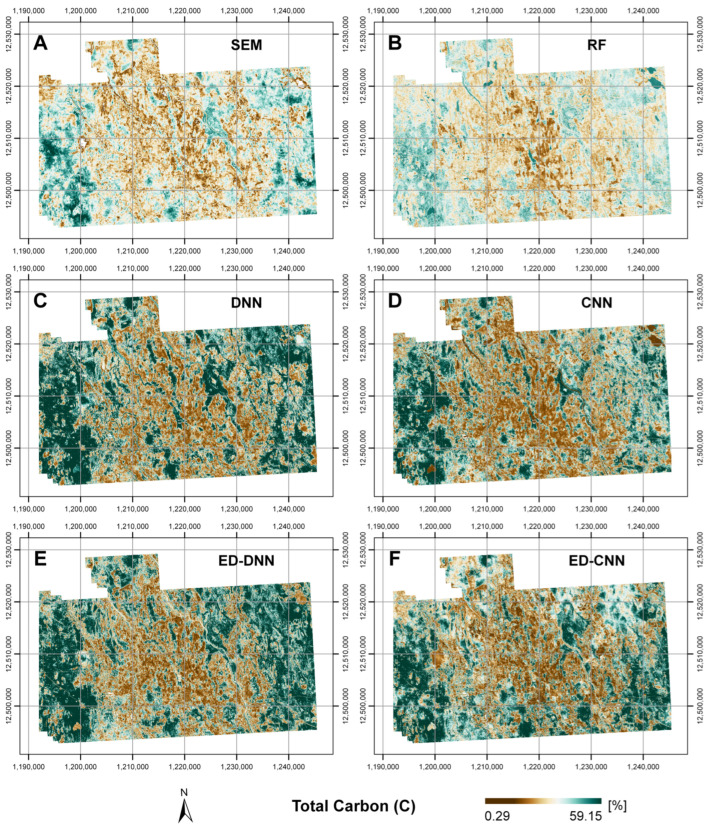
Prediction maps of total carbon (C) [%] for each respective model for the Cochrane study area. These prediction maps correspond to (**A**) random forest (RF), (**B**) structural equation modeling (SEM), (**C**) dense neural network (DNN), (**D**) 1-D convolutional neural network (CNN), (**E**) encoder-decoder composed of dense neural network layers (ED-DNN), and (**F**) encoder-decoder composed of 1-D convolutional neural network layers (ED-CNN). Coordinate grids are in the NAD 1983 Lambert conformal conic projection, with eastings [m] and northings [m] marked.

Prediction maps of soil C for each model for the Cochrane study area are displayed in Figure 6. These correspond to the SEM, RF, DNN, CNN, ED-DNN, and ED-CNN models. All maps are depicted with the same symbology settings for total carbon (C) [%], with the lowest C contents denoted by brown coloring and the highest C contents with green coloring as featured by the scale bar in the legend.

Regarding Figure 6, similar trends were observed for all the prediction maps. In general, land covers corresponding to agricultural fields had the lowest C, and conversely, wetlands had the greatest C. The prediction map for the ED-CNN (Figure 6F) conformed to the highest accuracy model (see Table 3). Predictions from the NNs (Figure 6C–F) had similar magnitudes for wetlands, whereas the SEM and particularly RF underpredicted C for the wetlands. As well, compared with other models the SEM and RF tended to overpredict C for localities surrounding the agricultural fields, such as for secondary forest and pasturelands. The models incorporating convolutional layers (Figure 6D,F) depicted localities with higher and lower predictions, as well as intermediate values; these discerned prediction for forested regions outside of the wetlands with lower but still relatively high C contents.

**Figure 7:**
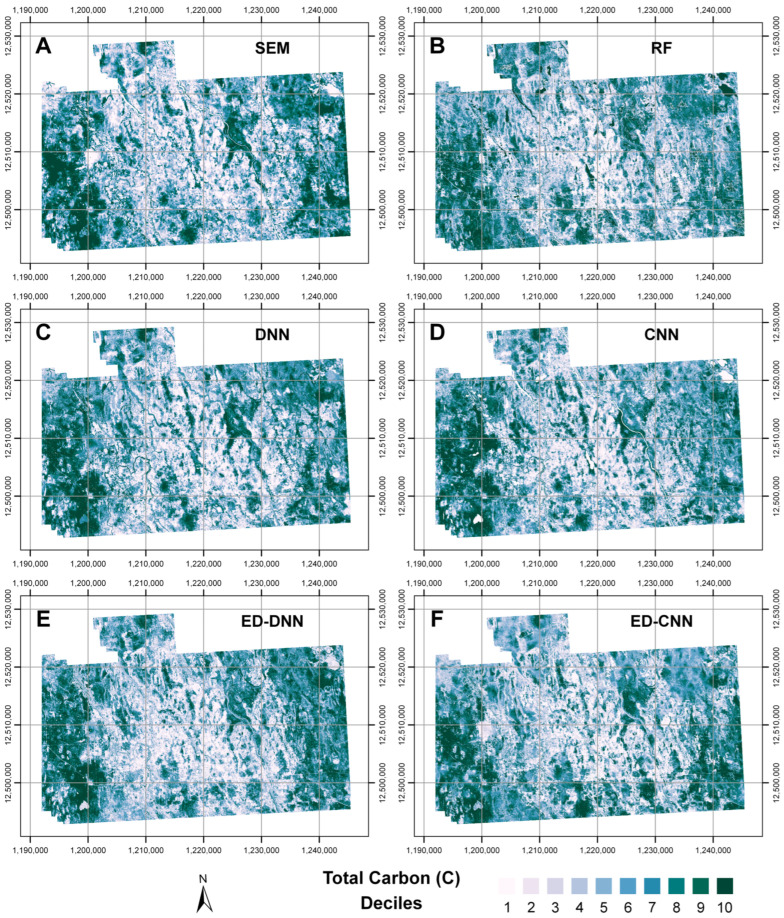
Prediction maps of deciles for total carbon (C) for each respective model for the Cochrane study area. These prediction maps correspond to (**A**) random forest (RF), (**B**) structural equation modeling (SEM), (**C**) dense neural network (DNN), (**D**) 1-D convolutional neural network (CNN), (**E**) encoder-decoder composed of dense neural network layers (ED-DNN), and (**F**) encoder-decoder composed of 1-D convolutional neural network layers (ED-CNN). Coordinate grids are in the NAD 1983 Lambert conformal conic projection, with eastings [m] and northings [m] marked.

Decile maps for each corresponding prediction map were also generated and are presented in Figure 7. The coloring intensity for the deciles was ordered, with lighter coloring corresponding to lower deciles accounting for lesser predicted soil C. Similar patterns with prediction are revealed in all these subfigures, indicating that in general, each model predicted C with similar trends to the various cover types, even if the magnitudes differed. Overall, the wetland areas were predicted to have the greatest C portions, with agricultural lands predicted to have the least C. These decile maps indicated that the models predicted relatively comparably between one another for various cover types, when mitigating for the tendency of models to overpredict C for localities with the most C and underpredict C for the areas with the most C, accordingly.

**Figure 8:**
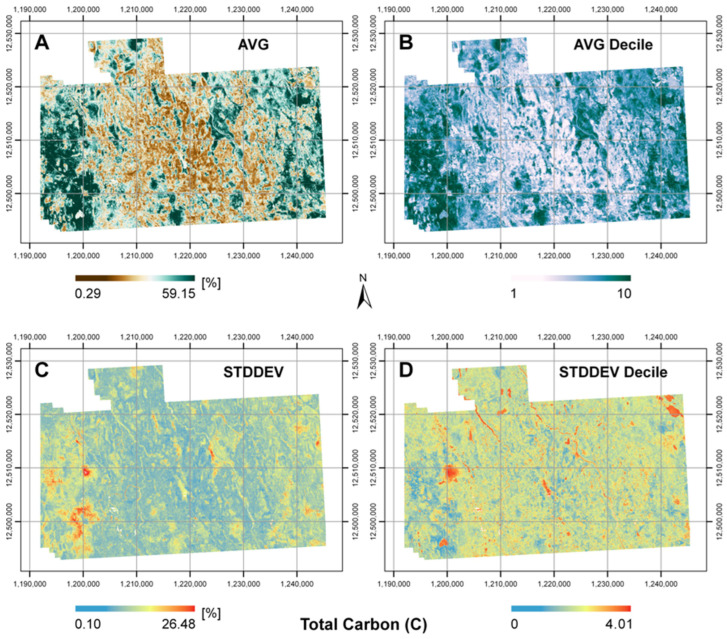
Ensembled prediction maps for the Cochrane study area. These prediction maps correspond to (**A**) mean of total carbon (AVG) [%], (**B**) mean of decile total carbon (AVG Decile), (**C**) standard deviation of total carbon (STDDEV) [%], and (**D**) standard deviation of decile total carbon (STDDEV Decile). Coordinate grids are in the NAD 1983 Lambert conformal conic projection, with eastings [m] and northings [m] marked.

The mean and associated standard deviation prediction maps are presented in Figure 8. Inspecting the standard deviation plot for C [%], the largest standard deviations appeared for the wetland localities. Although wetlands were consistently predicted to have the highest C, the magnitude of prediction varied, which resulted in high standard deviations. The lowest standard deviations were for the cropland areas, which contained the lowest C. When examining the standard deviation plot generated from the decile maps, the wetlands did not exhibit as great of standard deviation values. Now, higher standard deviations were revealed for forested regions, particularly for areas next to streams or on the peripherally of wetlands. On that account, both types of standard deviation maps were useful as they conveyed supplemental information. When deciles were considered, the regions of highest modeling uncertainties were no longer the wetlands but regions of forest that were likely more diverse with vegetation.

## 4. Discussion

The quantities of target data here for soil mapping, as is common in soil research studies [4], restricted the complexity of NNs employed in mitigating the effects of overfitting models. Consequently, the NNs were formulated to consist of fewer layers and units, to minimize the number of parameters to train. This was partly accomplished ad hoc and by consulting similar formulations for regression [30], before settling on the final numbers of units for the hidden layers of the NNs. DNNs and CNNs of fewer units per layer were investigated, specifically with no more than 16 units per hidden layer. However, the attained modeling accuracies were not as great as those attained when up to 32 units per hidden layer were set. As noted in Table 2, the number of parameters trained corresponded to 1784 for the encoder and 833 for the decoder for the ED-DNN and 1368 for the encoder and 3729 for the decoder for the ED-CNN, respectively. Likewise, the DNN and CNN models reported in Table 3 required the training for 1665 and 2721 parameters, accordingly. For perspective, this study consisted of 144 sampling sites, where 103 were allocated for model training. Thus, reasonable specifications were set for the number of units for hidden layers for NNs in this study. It is not uncommon for CNNs that are employed for regression training to require the optimization of tens of thousands, if not over a million, parameters [18]. The EDs presented in this study effectively augmented modeling accuracies for smaller datasets.

An alternative process for normalizing the predictor data was also investigated. A simpler data normalization enacted by linearly scaling each predictor between respective minimums and maximums, for values to correspond between 0 and 1, was also applied [54]. However, this normalization impacted the stability of the NNs as modeling accuracy notably degraded between simulation runs or when a few additional epochs were added for training. It was crucial to suitably normalize predictor data for training with the NNs, so consequently, standardization by z-score [18] normalization was implemented. In this study, means and standard deviations for each predictor were resolved from the whole input data set. These same means and standard deviations were used to calculate z-scores [20] for both the training and validation sets. This was necessary as the NNs were trained on z-score values for the predictors, so hence, the predictor values were transformed similarly on the validation set for model assessment. Moreover, these same means and standard deviations also needed to be adopted for normalizing predictor layers for the subsequent generation of prediction maps; otherwise, predictor values would not directly compare with those wielded for model training. On that account, these are important considerations for transforming predictor values to z-scores when prediction maps were an ultimate aim with the modeling.

Regarding modeling accuracy, the ED models were a definite improvement from the respective DNN and CNN models, as indicated in Table 3. These ED models appeared to filter out some of the noise within the predictor data and unravel nonlinear interactions between the predictors that would have been difficult to reveal with conventional regression approaches. In digital soil mapping applications, it is typical for dozens of predictors to be incorporated into a model, even when only a few of those predictors explicate the overwhelming majority of prediction power [4]. It is likely that additional predictors do contribute some information gain for certain attributes such as types of land cover, but this information is lost as noise within model training. Furthermore, differences exist for the functioning between each of the ED-DNN and ED-CNN. ED-CNNs are capable of successfully integrating NN feature extraction and ED structure for sequential data [27]. CNNs can extract features from localized regions, from which crucial features can be condensed into an abstract representation via an encoder and from which the decoder subsequently transforms into the target sequence for output [27]. The ED-DNN can handle nonlinearity or nonconvexity within data [29], without encapsulating local features within input data as resolved by convolutional layers [27]. However, an ED-DNN typically requires the optimization of fewer parameters during training versus that for an ED-CNN, so ED-DNNs can be preferrable for regression for smaller datasets.

The simplest type of CNN, i.e., composed of 1-D convolutional layers, was utilized for the CNN training. As the target data, here C, conformed to data points, only a 1-D CNN corresponding to predictors for a site was adopted for the ED-CNN and CNN models. Alternatively, 2-D CNNs, i.e., CNNs consisting of 2-D convolutional layers, have been commonly applied on imagery [18] for model training. In addition to a finite number of soil sampling sites, the spatial extent of each sampling site was typically less than 20 m^2^. This meant that a sampling site conformed to only 1 pixel of Landsat-8 imagery, of 30 m cell size. Unless finer spatial resolution imagery was obtainable for these sampling sites for all predictors, only one pixel of imagery would be inputted, constraining the extent of imagery constituting a site. For comparison’s sake, a basic 2-D CNN was trained for modeling C with these predictors. This CNN utilized the same layer formulation as the 1-D CNN but replaced with 2-D convolution layers. When fitted, this CNN required the optimization of 18,561 parameters and attained an R^2^ of 0.26 on the validation set, which was worse than that of the 1-D CNN reported in Table 3. Based in part on these reasons, only CNNs composed of 1-D convolution structure were employed.

Imbalances between training and validation datasets, which can arise in part due to sampling site locations that are spatially clustered, can lead to models attaining accuracies that are optimistic. This occurrence can be mitigated regarding the cross-validation approach implemented. With k-folds cross-validation [55,56] the number of folds specified can range from a minimum of two, corresponding to a single training and validation set split. On the contrary, the maximum number of folds can equal the number of sites, which conforms to a leave-one-out cross-validation (LOOCV) [55]. Typically, per iteration the models are trained and calibrated on k-l folds, with the remaining fold set aside for assessing model predictions [55]. Commonly, 5-fold or 10-fold cross-validations are adopted [56]. For datasets of limited sizes, an LOOCV can be appropriate as this can decrease modeling bias [56]. However, increasing the number of folds necessitates equivalently longer training times and computational resources as models need to be re-calibrated for each iteration of the k-folds. This can require that for each iteration, the trained model must be saved so that modeling accuracies can be averaged over all the trained models from across the folds. Moreover, this is also an issue for generating prediction maps, which can entail the averaging of predictions from the trained models for each iteration. In a related manner, there are also spatial considerations regarding cross-validation. Nevertheless, spatial cross-validation approaches can impel misconceptions with statistical validity [55], so validation with regard to probability sampling via statistically rigorous approaches is preferable [55]. For instance, stratified random sampling with respect to the targeted modeling property can address imbalances between datasets arising from splits.

Uncertainty with prediction is an important issue to address, particularly within digital soil mapping [4]. For C modeling in the boreal context, it is well established that wetlands comprise the greatest quantities of C [4]. However, due to the greater concentration of C within wetland sites, it can be difficult to train models that operate equally well over all land cover types. Thus, any complementary information obtained, even in the context of uncertainty mapping, is of use for interpretation purposes. The output of prediction maps from the different modeling approaches can be ensembled for standard deviation calculations. This imparts what land cover types have the greatest discrepancies with prediction. Additionally, the consideration of quantile maps for standard deviation calculations can disclose useful knowledge, when accounting for outliers or overprediction and underprediction. In this study, uncertainty mapping via decile maps in Figure 8 revealed that greater amounts of modeling uncertainty prevailed for sites with intermediate values of soil C and not just for wetlands. This indicated a need for predictors that better encapsulated characteristics for sites with intermedial C concentrations.

On that note, limitations with the modeling can be attributed in part to the predictors wielded. Inspecting the scatterplots in Figure 4, the residual patterns displayed between the models are consistent with one another. Although the predictors utilized for modeling were retrieved from various remote sensing technologies and conformed to different soil formation factors, the inclusion of other distinctive environmental covariates for predictors could be investigated. The structure of an ED can effectively filter out noise within predictors, discerning useful information from large sets of predictors. Therefore, additional predictors can be incorporated into modeling. As previously mentioned, modeling can be enhanced by the consideration of features relating to varying concentration levels of soil C. Observing the scatterplots from Figure 5, the best model overpredicted C for sites with low C contents but underpredicted C for the sites with the greater C concentrations. In boreal regions, wetlands tended to contain the highest C content due to their composition of peat [4]. However, differentiating the classification between wetlands and old growth forest can be difficult to ascertain, especially if both cover types are composed of black spruce (*Picea mariana*) [57]. All in all, there is an advantage to collecting diverse types of environmental covariates to employ as predictors, particularly if EDs can extract practical information from higher-dimensional datasets.

Interpretating the effects of predictors with modeling is of importance, especially in quantifying interactions between these features with respect to soil formation factors. This is an additional interest to research, in conjunction with improving the accuracies of soil modeling. Accuracies pertaining to digital soil mapping for boreal regions within Canada tend to be relatively low [14,15], so enhancing these accuracies can conceivably be achieved through both EDs and the application of novel environmental covariates. Other modeling approaches, such as via variable importance with RF and path analysis with SEM, can assist with unfolding relationships between environmental covariates and targeted soil properties. A future research direction relates to the synergy of deeper learning approaches with more conventional approaches such as SEM, particularly in expounding relations between predictors in connection with attributes of soil properties in boreal study regions. In tandem with deeper learning is the pursuit of implementing attention mechanism and skip connection architecture [58,59] to increase weightings for the important features. All in all, this can potentially contribute to enhancing the modeling of soil properties for land covers with higher uncertainties with prediction, such as for forested sites composed of various tree species.

## 5. Conclusions

ED approaches implementing NNs suitable for smaller data sets were investigated to enhance modeling accuracies for the regression of soil C in a boreal study area within northern Ontario, Canada. These consisted of ED-DNN and ED-CNN models, which were also compared with DNN, CNN, RF, and SEM models. The ED-CNN attained the best modeling accuracies, with an R^2^ of 0.59 on the validation set. Prediction maps were generated from all these approaches, which were ensembled together to obtain uncertainty estimates for prediction. In addition, decile maps for each model were computed, from which a map corresponding to standard deviations of the deciles was constructed. For land cover, wetlands had the highest uncertainties with prediction for the standard deviation map in retrieved C units [%]. Contrarily, land covers with intermediate C concentrations depicted the highest uncertainties in the standard deviation map conforming to the deciles. These insights will support the selection of environmental covariates for soil formation factors more applicable for differentiating forest cover with lower or higher C concentrations, for future digital soil mapping applications.

## Figures and Tables

**Table 1:** Listing of predictors wielded for modeling.

Predictor	Source	Soil Formation Factor
Digital elevation model (DEM) *	LiDAR	Relief
Canopy height model (CHM)	LiDAR	Vegetation
Gap fraction	LiDAR	Vegetation
Aspect	DEM (LiDAR)	Relief
Convergence index	DEM (LiDAR)	Relief
Mid-slope position *	DEM (LiDAR)	Relief
Multi-resolution ridge top flatness (MRRTF)	DEM (LiDAR)	Relief
Multi-resolution valley bottom flatness (MRVBF)	DEM (LiDAR)	Relief
SAGA topographic wetness index (SAGA TWI)	DEM (LiDAR)	Relief
Slope *	DEM (LiDAR)	Relief
Slope height *	DEM (LiDAR)	Relief
Slope length	DEM (LiDAR)	Relief
Stream power index	DEM (LiDAR)	Relief
Terrain ruggedness index (TRI)	DEM (LiDAR)	Relief
Topographic wetness index (TWI)	DEM (LiDAR)	Relief
Total curvature	DEM (LiDAR)	Relief
Valley depth	DEM (LiDAR)	Relief
Visible sky	DEM (LiDAR)	Relief
B1 summer 2017	SR	Vegetation
B2 summer 2017	SR	Vegetation
B3 summer 2017	SR	Vegetation
B4 summer 2017	SR	Vegetation
B5 summer 2017	SR	Vegetation
B6 summer 2017	SR	Vegetation
B7 summer 2017	SR	Vegetation
B10 summer 2017	SR	Vegetation
Modified normalized difference water index (MNDWI) summer 2017	SR	Relief (water)
Normalized difference vegetation index (NDVI) summer 2017	SR	Vegetation
Change magnitude B3 B4 B5 summer 1984–2005 *	SR	Time
SAR C VH May 2017	SAR	Relief (water)
SAR C VV May 2017 *	SAR	Relief (water)
Gravity anomaly 2016	Aeromagnetic	Parent material
Magnetic residual November 2018	Aeromagnetic	Parent material
NFI black spruce 2011 *	k-NN model	Vegetation

* Predictor for SEM.

**Table 2:** Encoder-decoder structures composed of dense neural network (ED-DNN) and 1-D convolutional neural network (ED-CNN) layers. Number (#) of parameters for each layer are also noted.

ED-DNN				ED-CNN		
Layer	Output Shape	# Parameters		Layer	Output Shape	# Parameters
Encoder:				Encoder:		
Input layer	34	0		Input layer	(1, 34)	0
Dense	32	1120		Convolution 1-D	(1, 16)	560
Dense	16	528		Max pooling 1-D	(1, 16)	0
Dense	8	136		Convolution 1-D	(1, 32)	544
Decoder:				Flatten	32	0
Input layer	8	0		Dense	8	264
Dense	32	288		Decoder:		
Dense	16	528		Input layer	8	0
Dense	1	17		Dense	64	576
				Reshape	(1, 64)	0
Total parameters:	2617			Convolution 1-D	(1, 32)	2080
				Upsampling 1-D	(2, 32)	0
				Convolution 1-D	(2, 16)	528
				Flatten	32	0
				Dense	16	528
				Dense	1	17
				Total parameters:	5097	

**Table 3:** Modeling accuracies for each model as assessed in the validation set. The reported metrics are coefficient of determination (R^2^), root mean squared error (RMSE), and mean absolute error (MAE).

Model		R^2^	RMSE	MAE
			[%]	[%]
Structural equation model	SEM	0.21	10.18	7.63
Random forest	RF	0.22	10.09	7.83
Dense neural network	DNN	0.18	10.38	7.35
Convolutional neural network 1-D	CNN	0.37	9.07	6.05
Encoder-decoder DNN	ED-DNN	0.54	7.78	5.40
Encoder-decoder CNN 1-D	ED-CNN	0.59	7.30	5.38
Ensemble *	AVG	0.50	8.06	6.11

* Average prediction of all six models.

## Data Availability

The original data presented in the study are openly available in FigShare at dx.doi.org/10.6084/m9.figshare.28250750.
